# Computed Tomography's Impact on the Surgical Planning for Posterior Malleolar Fractures

**DOI:** 10.1055/s-0045-1810040

**Published:** 2025-09-08

**Authors:** Noé De Marchi Neto, Pietro Felice Tomazini Nesello, Jordanna Maria Pereira Bergamasco, Marco Túlio Costa, Ralph Walter Christian, Nilson Roberto Severino

**Affiliations:** 1 Department of Orthopedics and Traumatology, Santa Casa de São Paulo – Pavilhão Fernandinho Simonsen, Faculdade de Ciências Médicas da Santa Casa de São Paulo, São Paulo, SP, Brazil

**Keywords:** ankle fractures, radiography, surgeons, tomography, x-ray computed, cirurgiões, fraturas do tornozelo, radiografia, tomografia computadorizada por raios x

## Abstract

**Objective:**

This study aimed to evaluate the influence of computed tomography (CT) on the preoperative planning of posterior malleolus (PM) fractures of the ankle, comparing its information with that of conventional radiographs and assessing its impact on surgical treatment.

**Methods:**

The study included 81 patients with PM fractures, whose radiological and CT images were analyzed by 33 specialized orthopedic surgeons. The study had two stages, with a radiological assessment on the first, and the second having radiological plus CT evaluation. In both stages, we asked surgeons about the PM size, fracture stability, preoperative management, and potential modifications after CT analysis.

**Results:**

Considering only radiographs, 83.5% of the evaluators selected PM fixation. However, CT addition modified this choice in 49.1% of the cases, influencing the surgical access route and the type of osteosynthesis. In 34.7% of cases, CT revealed a larger PM fragment than radiographs, demonstrating that it is superior in evaluating fracture size and morphology.

**Conclusion:**

The surgical planning of ankle fractures with PM involvement should routinely include CT scans for a more precise fracture line assessment and a potential change in the therapeutic decision based on simple radiography alone.

## Resumo

**Objetivo:**

Este estudo teve como objetivo avaliar a influência da tomografia computadorizada (TC) no planejamento pré-operatório das fraturas do maléolo posterior (MP) do tornozelo, comparando suas informações com as de radiografias convencionais (RX) e o seu impacto no tratamento cirúrgico.

**Métodos:**

A pesquisa incluiu 81 pacientes com fraturas do MP, cujas imagens de RX e TC foram analisadas por 33 cirurgiões ortopédicos especializados. O estudo foi dividido em duas etapas, com avaliação de RX na primeira e de RX e TC na segunda. Em ambas as etapas, perguntou-se o tamanho do MP, a estabilidade da fratura, a conduta pré-operatória e as possíveis modificações após avaliar a TC.

**Resultados:**

Os resultados mostraram que, ao considerar apenas o RX, 83,5% dos avaliadores decidiram pela fixação do MP. No entanto, ao adicionar a TC, essa decisão foi modificada em 49,1% dos casos, influenciando a via de acesso cirúrgico e o tipo de osteossíntese. A TC revelou, em 34,7% dos casos, um fragmento maior do MP em comparação ao RX, demonstrando sua superioridade na avaliação do tamanho e morfologia da fratura.

**Conclusão:**

A TC deve ser incluída de forma rotineira no planejamento cirúrgico das fraturas do tornozelo com comprometimento do maléolo posterior, pois permite uma avaliação mais precisa do traço de fratura e pode mudar a decisão terapêutica baseada somente na RX.

## Introduction

Ankle fractures are common in orthopedic practice. Approximately 40% of malleolar ones involve the posterior malleolus (PM). This type of fracture results in greater instability and joint incongruence, higher complexity in joint reduction, and a higher long-term risk of developing osteoarthritis. 1 2

For many years, PM fracture treatment relied upon preoperative radiographs showing the lesion size regarding the tibiotalar joint. 3 However, radiographic PM evaluation has been criticized since the end of the 20 ^th^ century as it may not clarify lesion complexity or lead to an underestimation of the posterior fragment size. 2 4 5 6 Starting in the 2000s, computed tomography (CT) has become a valuable assessment tool for PM fractures, providing a more accurate interpretation of fracture patterns and assisting preoperative planning due to the introduction of new classifications for the posterior fragment alone. 7 8 9

Preoperative CT scans reveal a larger PM than radiographs, 10 and provide more information regarding fracture lines, joint depression, interposed fragments, as well as the relationship between PM, other malleoli, and syndesmosis. The current recommendation for the surgical planning of ankle joint fractures is the combined use of CT and radiological evaluation. 5 11 12

This study aimed to assess the impact of CT scans on the preoperative evaluation of ankle fractures and how CT information influences PM interpretation and treatment selection by orthopedic surgeons.

## Materials and Methods

Over 5 years, from 2016 to 2021, 144 patients with ankle fractures involving the PM underwent treatment at our Hospital's Level 1 Trauma Center. We selected cases of ankle fractures or fracture-dislocations involving the PM in patients over 18-years-old and with adequate radiological and CT documentation for the study. We excluded subjects with tibial pilon fractures, ankle fractures with other hindfoot fractures, and immature skeletons. The Research Ethics Committee analyzed and approved this study under the CAAE number 52916921.1.0000.5479.

**Fig. 1:**
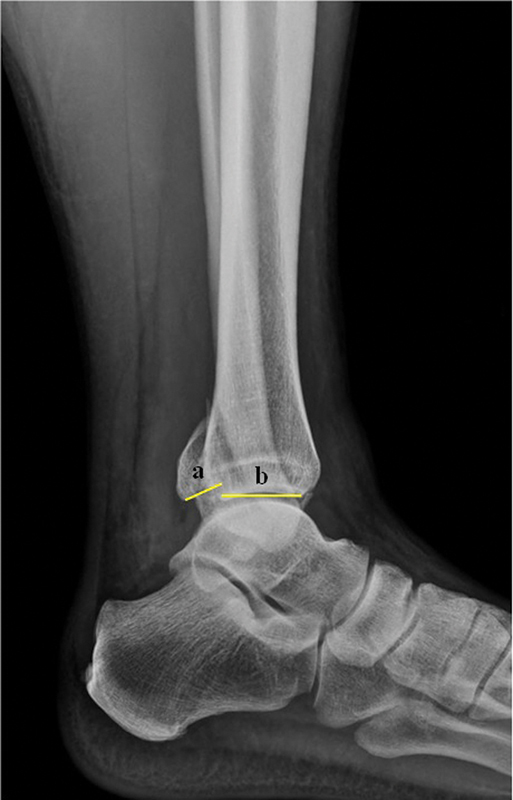
Measurement of the posterior malleolus fracture fragment size on a lateral radiograph. The proportion between the fracture and the entire articular surface was determined by the ratio (a)/(a + b).

The study included 81 patients, who underwent a pre-evaluation by two orthopedists from our institution. Both were specialists in foot and ankle surgery, and independently measured PM dimensions in imaging tests ( Figure 1 ) to determine the fracture's classification per the Haraguchi system. 7 We defined the average percentage size of the PM fragment over the total articular surface and the CT-based classification as standard values.

Using the standard values, we selected cases to form sets of 10 PM fractures with different radiological sizes. The ranges included <15.0%, from 15.0 to 19.9%, from 20.0 to 24.9%, from 25.0 to 29.9%, and >30.0% of the total articular surface, as well as the different tomographic PM fracture types.

The study had two stages, with the order of the cases presented to the evaluators being randomized for each stage ( Table 1 ).

**Table 1:** Significance of PM in ankle fracture stability according to its size on radiography

Cases	Stability	Mean	SD	Inferior 95% CI	Superior 95% CI	*p* -value
< 15%						
	No	79.4%	30.4%	68.6%	90.2%	< 0.01*
	Yes	20.6%	30.4%	9.8%	31.4%	
15–19.9%						
	No	45.6%	39.6%	31.5%	59.6%	< 0.01**
	Yes	54.4%	39.6%	40.4%	68.5%	
20–24.9%						
	No	44.1%	29.6%	33.6%	54.6%	< 0.01***
	Yes	55.9%	29.6%	45.4%	66.4%	
25–29.9%						
	No	11.8%	30.3%	1.0%	22.5%	< 0.01***
	Yes	88.2%	30.3%	77.5%	99.0%	
> 30%						
	No	14.7%	35.9%	2.0%	27.5%	NA
	Yes	85.3%	35.9%	72.5%	98.0%	

**Abbreviations:** CI, confidence interval; NA, not available; PM, posterior malleolus; SD, standard deviation.

**Notes:** *Comparison with all other PM categories. **Comparison with 25–29.9 and ≥ 30% PM categories. ***Compared with > 30% PM category.

**Fig. 2:**
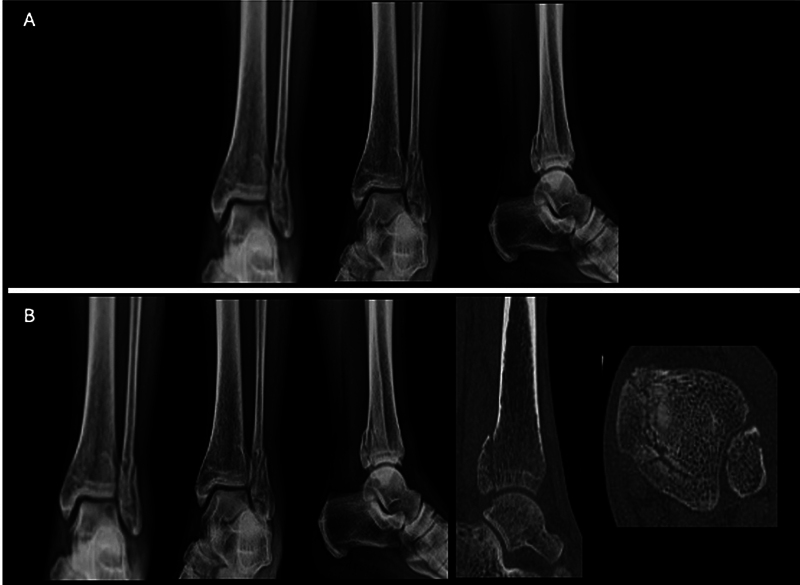
Example of a case sent to each evaluator. Stage 1, with ( **A** ) radiograph alone, and ( **B** ) with radiograph, sagittal computed tomography (CT) section with the largest posterior malleolus fragment, and axial CT section 5 mm above the articular surface.

For the first stage (S1), we organized the cases alphabetically, from A to J, using ankle radiographs in anteroposterior and lateral views with emphasis on the PM fracture. For the second stage (S2), we reorganized the same cases from Q to Z, presenting the same radiographs as S1, now complemented by sagittal and axial CT images detailing the fracture ( Figure 2 ).

We invited 40 orthopedic surgeons from different hospitals to participate as evaluators. Those who accepted to participate in the study completed a consent form approved by the institution's Medical Ethics Committee. To participate, each evaluator had to be a specialist with training in foot and ankle surgery, affiliated with a Medical Residency Teaching Service in Orthopedics and Traumatology, or a Training Center for Specialists in Foot and Ankle Surgery with accreditation by the respective Societies.

The evaluators received a set of 10 cases by email and answered individually to the two stages of the survey, with a 2-month interval between them. Each case provided information on was provided on age, gender, and trauma mechanism (ankle sprain, fall from a height, traffic accident, and trauma during sports practice). Additionally, each set included a standardized questionnaire. The S1 questions addressed radiographic imaging alone, while S2 questions were about radiological and CT scan ( **Annex 1, Questionnaire** ).

In both stages, radiological questions addressed aspects such as fracture stability, interpretation of the PM size, fixation indication, and recommended fixation type. The CT scan questions included comparisons of the PM size about radiography, tomographic classification, potential changes in treatment, fixation indication, and synthesis type for use.

After completing the two stages, we compared S1 and S2 answers regarding radiographs to assess intra- and interobserver agreement at two different times. The analysis of CT scan answers verified the tomographic classification accuracy by the evaluators and underwent a comparison with the responses given to the radiographic questions. Lastly, we assessed the extent to which CT scans influenced fracture interpretation and defined the preoperative planning.

## Statistical Analysis

Data analysis used the Statistical Package Social Sciences (SPSS, IBM Corp.), version 25 for MAC software. We described categorical data as absolute and relative frequencies. The kappa and McNemar tests assessed intra- and interevaluator agreement.

We described continuous or numerical data as mean and standard deviation (SD) values and, when appropriate, median and 25 ^th^ and 75 ^th^ percentiles. The Shapiro-Wilk test determined continuous data normality. To compare the proportions of therapeutic changes, we used the following nonparametric tests: Mann-Whitney's for independent samples and Friedman's for multiple comparisons.

All statistical analyses considered a *p* < 0.05 significance level.

## Results

The study included 33 evaluators with a mean age of 45 ± 8 years. Their mean experience time in Foot and Ankle surgery was 15 ± 7 years, and they treat an average of 65 (39–86) ankle fractures per year.

## Posterior Malleolus Size

Radiographic analysis revealed that an average of 59.7 ± 15.3%) of the evaluators estimated the PM size similarly to the standard values from the subgroups, with concordance rates of 0.16 and 0.15, respectively, in the two stages. In S1 and S2, 78.8 ± 19.9% of the evaluators considered the PM size relevant for fracture stability, and its importance increased proportionally to the size of the fragment, with a concordance rate of 0.21 ( *p* < 0.01), as shown in Table 1 .

Considering CT scans alone, evaluator agreement regarding the Haraguchi 7 classification was 66.1 ± 4.82%). Moreover, in 60.9 ± 15.8%) of the cases, the evaluators indicated a PM size on the CT scan similar to the one they had measured on radiographs.

**Fig. 3:**
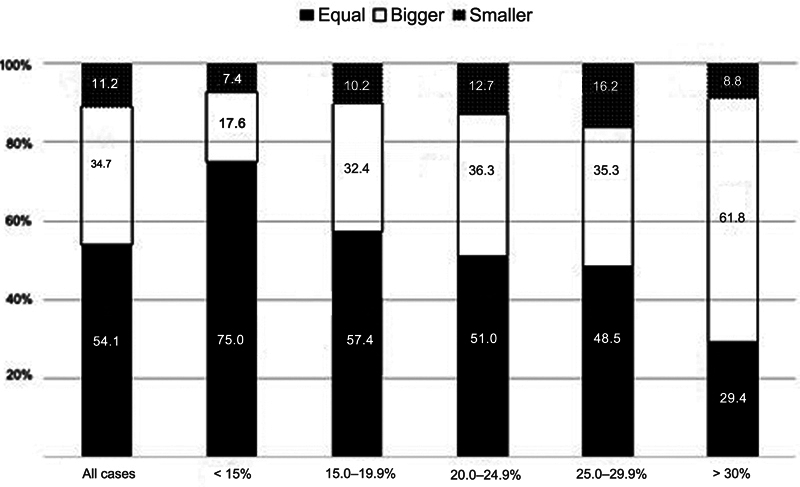
Evaluator interpretation when comparing the posterior malleolus size in radiographs and CT scans.

At the simultaneous comparison of PM size on radiographs and CT scans, 54.1% of the evaluators considered the sizes similar in both imaging modalities. The PM was considered larger on CT scans than on radiographs in 34.7% of the cases, and smaller on CT scans in 11.2% fractures. Table 2 and Figure 3 illustrate the differences in PM sizes between the subgroups. We can see that, as the size increases, the greater the divergence in interpretation between radiographs and CT scans.

**Table 2:** Comparison of the PM size on CT with the previous radiological measurement in all cases and at each interval

	Equal		Bigger		Smaller		
Radiological PM size	Mean	SD	Mean	SD	Mean	SD	*p* -value
**Total**	54.1%	16%	34.7%	16%	11.2%	11%	NA
**< 15%**	75.0%	30.8%	17.6%	29.9%	7.4%	18.0%	< 0.05*
**15–19.9%**	57.4%	35.1%	32.4%	32.3%	10.3%	23.9%	< 0.05**
**20–24.9%**	51.0%	33.1%	36.3%	30.0%	12.7%	16.4%	> 0.05***
**25–29.9%**	48.5%	33.7%	35.3%	33.8%	16.2%	29.4%	NA
**> 30%**	29.4%	46.2%	61.8%	49.3%	8.8%	28.8%	< 0.05*

**Abbreviations:** CT, computed tomography; NA, not available; PM, posterior malleolus; SD, standard deviation.

**Notes:** *Comparison with all PM categories. **Comparison with 20–24.9% and 25–29.9% PM categories. ***Comparison with 25–29.9% PM category.

## Posterior Malleolus Treatment

Evaluating radiographs alone, 83.5 ± 11%) of the evaluators agreed on PM fixation (or not) in S1 and S2, with respective agreement rates of 0.36 and 0.34.

**Fig. 4:**
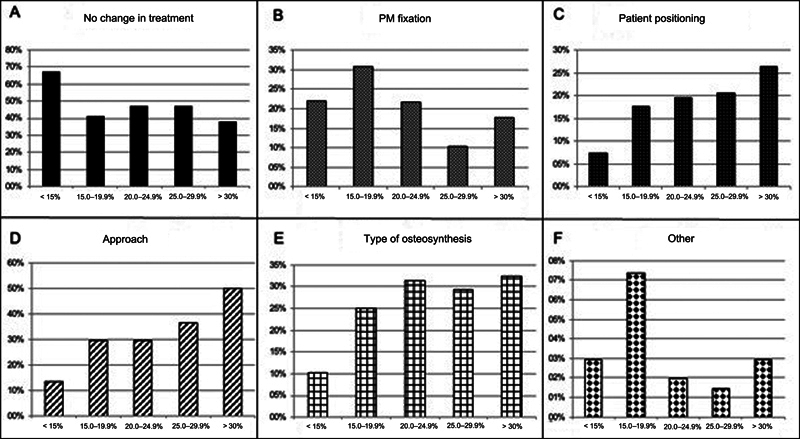
Changes in the evaluators' treatment after the combined assessment of radiography and CT findings. ( **A** ) No treatment change, ( **B** ) PM fixation after seeing the CT scan, ( **C** ) patient positioning modification during surgery, ( **D** ) surgical approach change, ( **E** ) type of osteosynthesis selected for fixation, ( **F** ), other modifications.

In the simultaneous analysis of radiographs and CT scans, the evaluators' treatment would not change in 49.1% of cases. In the remaining cases, the CT scans influenced the clinical decision, changing the approach in 29.7%, the osteosynthesis type in 25.6%, the indication for PM fixation in 20.9%, and the decubitus position in 17.6% of cases. Table 3 presents the suggested treatment changes for each subgroup, and Figure 4 illustrates these modifications. In groups with fractures involving less than 25% of the PM, the most frequent change was indicating fixation. In fractures with fragments larger than 25%, there was a progressive increase in therapeutic changes regarding approach, positioning, and osteosynthesis selection.

**Table 3:** Modifications in evaluator treatment due to different PMsizes after the combined analysis of radiograph and CT findings

PM size	None	PM fixation	Patient positioning	Approach	Osteosynthesis type	Other
< 15%	67.6%	22.1%	7.4%	13.2%	10.3%	2.9%
15–19.9%	41.2%	30.9%	17.6%	29.4%	25.0%	7.4%
20–24.9%	47.0%	21.5%	19.6%	29.4%	31.3%	1.9%
25–29.9%	47.0%	10.3%	20.5%	36.7%	29.4%	1.4%
> 30%	38.2%	17.6%	26.5%	50.0%	32.4%	2.9%

**Abbreviations:** CT, computed tomography; PM, posterior malleolus.

## Discussion

The CT scan plays a significant role in preoperative planning of PM fractures due to three main factors. First, it allows accurate visualization of the actual size of the posterior fragment. 10 Second, radiographs alone cannot properly evaluate fractures extending beyond the posterolateral portion of the tibia or with multiple or interposed fragments. 13 14 Finally, CT scans assist the surgeon in selecting the approach, patient positioning, and fixation implants. 12 15 Today, many authors recommend its routine use preoperatively in ankle fractures. 5 16

In this study, analysis of radiographic images revealed a significant variation in the accuracy of evaluators when measuring PM size, with an average of 59.7 ± 15.3% to standard values. Although this average was satisfactory, the agreement rates were low, at 0.16 and 0.15 in both stages. These results are consistent with other publications also reporting low agreement rates between evaluators analyzing radiographic parameters for PM fractures. 17

Most (78.8 ± 19.9%) evaluators emphasized the relevance of PM for fracture stability in S1 and S2. As expected, this recognition increases as the size increases, due to the traditional indication of fixation for fragments larger than 25%. However, the fixation rate for fragments smaller than 25% has grown, aiming to improve joint stability. Although the study highlighted the significance of the PM size, recent literature emphasizes that its morphology and correct reduction are crucial factors for treatment planning. 18 19 20

For CT images, evaluators presented an average accuracy rate of 66.1 ± 4.82% at the Haraguchi classification. 7 Our findings are consistent with comparative studies of the main tomographic classifications. 21 22 23 The Haraguchi classification was selected because it was the first to be described and it remains widely used, often combined with the Bartoníček classification. 8 21 22

The agreement rate for PM size in radiographs and CT scans was 54.1%. However, the size was larger on CT scans in 34.7% of cases. This difference increased in larger PMs, reinforcing the hypothesis that CT provides a more precise three-dimensional fracture analysis, especially those with higher complexity. 5 13 14 15 The increasing divergence between radiographic and CT measurements as PM increases suggests CT is better even for larger fragments, potentially with direct implications for treatment choice. 11

When assessing the posterior fracture treatment, most evaluators agreed with the decision to fix or not the PM based on radiographs alone (83.5%, with an agreement rate of 0.36). However, the introduction of CT changed the conduct in 49.1% of cases, influencing the choice of approach (29.7%), the type of osteosynthesis (25.6%), and the indication for PM fixation (20.9%). These findings reinforce the relevance of CT in preoperative evaluation and treatment planning. Gibson et al. demonstrated it significantly alters surgical planning in trimalleolar fractures, changing the surgical technique in 25.1% of cases, and a 16.3% trend to opting for fixation after CT scan analysis. 24

For treating PM fractures smaller than 25%, the main change was the increased indication for fixation, suggesting that radiography may underestimate the significance of PM in fracture stability, and that CT could offer a more detailed assessment by revealing the actual lesion size and potential joint deviations. 5 12 15 For fractures with fragments larger than 25%, size alone usually justifies fixation, but CT analysis resulted in significant therapeutic changes, including patient positioning, surgical approach, and type of osteosynthesis. These adjustments allowed a more accurate interpretation of the PM fragment by evaluators. 25

This is a pioneer study in Brazilian literature, relying on a long case series evaluated by 33 experienced orthopedists. However, it has some limitations. As a retrospective study focused exclusively on the PM fragment, it included no clinical or physical examination data from the patients. Furthermore, since the objective was to investigate the preoperative interpretation of the PM alone, the study does not address the treatment of each case or long-term functional results, limiting the analysis to clinical and therapeutic outcomes.

## Conclusion

This study reinforces the significance of CT in the preoperative planning of PM fractures. This imaging technique allows for a more precise analysis, especially in complex and multifragmented fractures, and it is essential to decide on the fixation for fragments smaller than 25%.

Moreover, even in fractures greater than 25%, CT allows meaningful adjustments in patient positioning, approach, and type of osteosynthesis, with direct impacts on surgical planning.

## Notes

Annex 1–Questionnaire
**STEP 1 (S1)**

1–Do you consider the PM size significant for the stability/instability of this ankle fracture?
  () Yes () No
2–Which is the PM size about the tibiotarsal joint?
  () smaller than 15% () from 15 to 19.9% () from 20 to 24.9% () from 25 to 29.9% () bigger than 30%
3–Would you perform PM fixation in this case based on the radiographic images?
  () Yes () No
4–Considering your indication for PM fixation, which surgical approach would you use?
  () Percutaneous () Lateral () Posterolateral () Medial () Posteromedial () Other () No fixation
5–What would you use for PM fixation?
  () K-wires () Cortical screws () Cannulated screws () Plate and screws () Other devices
6–In your interpretation, is radiograph enough to plan the treatment of this fracture or is some additional test necessary preoperatively? Which one?
  () Radiograph alone () CT () MRI () CT and MRI () Other tests

**STEP 2 (S2)**

1–Do you consider the PM size significant for the stability/instability of this ankle fracture?
  () Yes () No
2–Which is the PM size about the tibiotarsal joint?
  () smaller than 15% () from 15 to 19.9% () from 20 to 24.9% () from 25 to 29.9% () bigger than 30%
3–Would you perform PM fixation in this case based on the radiographic images?
  () Yes () No
4–If you would perform fixation, would approach would you use? *(Do not answer if you do not indicate fixation.)*
  () Percutaneous () Posteromedial () Posterolateral () Lateral () Medial
5–If you would perform fixation, what would you use for osteosynthesis? *(Do not answer if you do not indicate fixation.)*
  () K-wires () Cortical screws () Cannulated screws () Plate and screws () Other devices
6–What do you consider most significant in this PM fracture?
  () Size () Deviation () Syndesmosis instability () Joint congruence () Other
7–What is the tomographic classification (Haraguchi) of this fracture?
  () Type I () Type II () Type III
8–What is PM size to the tibiotarsal joint considering the radiographs and CT scan?
  () < 15% () 15–19,9% () 20–24,9%
  () 25–29,9% () > 30%
9–In your interpretation, when evaluating radiographs combined with CT, the PM size is:
  () Equal to what the radiography shows () Bigger () Smaller
10–When evaluating the CT, would you change your approach regarding the PM in this fracture?
  () Yes () No
11–What would CT make you change at the time of surgery regarding the PM?
  () Nothing () Indicate PM fixation () Patient positioning () Approach () Type of osteosynthesis () Other
  **Abbreviations** :
  CT – computed tomography;
  MRI – magnetic resonance imaging;
  PM – posterior malleolus.
